# The Trump presidency: Cascading global shocks on global health

**DOI:** 10.1371/journal.pgph.0005385

**Published:** 2025-11-26

**Authors:** Lawrence O. Gostin

**Affiliations:** O’Neill Institute for National and Global Health Law, Georgetown University Law Center, Washington, DC, United States of America; London School of Economics and Political Science, UNITED KINGDOM OF GREAT BRITAIN AND NORTHERN IRELAND

The world has been experiencing cascading global shocks, tightly interwoven with global health—the COVID-19 pandemic, climate change, conflicts, and humanitarian crises propelling mass migrations. On January 20, 2025, global health experienced a shock of another sort, the inauguration of Donald J. Trump.

On day one, President Trump issued an executive order giving notice of the U.S. intention to withdraw its membership in the World Health Organization (WHO) [1]. While the United States is legally bound to give a year’s notice and pay all debts, the president immediately stopped all funding and ordered the U.S. Centers for Disease Control and Prevention (CDC) to cease all communications with WHO. Another executive order that day froze all new obligations and disbursements of foreign assistance funds [2]. Days later, Secretary of State Marco Rubio issued a stop-work order, requiring nearly all ongoing foreign assistance activities to come to an immediate halt [3]. And that was only the first week.

Here and in an avalanche of other unilateral executive actions, the Trump administration has asserted a constitutional vision of the virtually unchecked powers of the presidency. The administration’s view of unfettered authority is at odds with the core constitutional principles of separation of powers and checks and balances, as well as with longstanding practice. Yet the U.S. Congress has not pushed back, and the Supreme Court has allowed most of the president’s executive actions to take effect as litigation challenging them plays out.

Here, I document key executive actions on global health taken by the Trump administration, and the destructive impacts on global health and U.S. national interests. The executive orders on withdrawing the United States from WHO and freezing foreign aid were most consequential to global health, and will be my primary focus. While numerous executive and legislative actions profoundly impact global health (e.g., climate change, immigration, tariffs, and biomedical research), they are largely beyond this article’s scope. I conclude with proposals for using this major rupture in international health cooperation as an opportunity to construct a more resilient global health financing and governance ecosystem. Out of peril there is opportunity for new alliances, self-reliance, and resilience *sans* the United States—at least until the next presidential election in 2028.

## The global health consequences

On September 18, 2025, Secretary Rubio published the *America First Global Health Strategy* [4]. “America First” is an oxymoron because global health requires global solidarity and international cooperation. And yet the strategy does enunciate what a U.S. populist global health agenda looks like: the core objective is to prevent diseases from abroad coming to U.S. shores; it elevates U.S. private sector and faith-based organizations; it shifts costs from U.S. taxpayers to partner countries; and it marginalizes international organizations and multilateral solutions. In the process, the America First agenda jettisons a long history of global health engagement.

### Disappearing global health leadership

When the United States decides to be a global health leader, it can lead like no other. With CDC and USAID already core partners in WHO-led initiatives such as eradicating smallpox and polio, as well as childhood immunization, U.S. global health engagement rose to a new level in 2003 with the launch of the President’s Emergency Plan for AIDS Relief (PEPFAR), the largest commitment by any nation to address a single disease. PEPFAR shows what is possible through compassionate, cost-effective, accountable, and transparent global health assistance.

U.S. health and humanitarian funding has been lifesaving. American contributions accounted for nearly half of all funding for humanitarian emergencies in 2024 – five times as much as the next largest funder, the European Commission [5]. The U.S. has provided one-third of Global Fund financing [6]. In the closing funding cycle, 2021–2025, the US provided 24% of the direct contributions to Gavi, the Vaccine Alliance [7]. All told, the US has been spending over $12 billion annually on global health, more than five times the contributions of the next largest funder, Germany [8].

Along with funding, the United States shares technical scientific expertise, above all through CDC, including emergency outbreak response, training, data collection, disease surveillance, and epidemiology. Last year, 1,700 CDC staff were based in more than 60 countries to surveil and respond to more than 400 diseases and health conditions [9]. The US launched the Global Health Security Agenda in 2014—now consisting of more than 70 partners that evaluate and strengthen health systems [10]. The Fogarty International Center at the National Institutes of Health (NIH) has trained 8,500 scientists from low- and middle-income countries (LMICs) [11]. And the Food and Drug Administration’s determination that a medicine is safe and effective is widely recognized as a gold standard, often used by LMICs in their own drug approval processes.

### Withdrawing from the World Health Organization

The U.S. has been WHO’s largest donor, contributing approximately 15% of its total budget [12]. U.S. contributions have funded some 25% of WHO’s Health Emergency Programme’s core work and 20% of its emergency response [13]. Due primarily to the halt in U.S. funding, WHO implemented a hiring freeze and major budget cuts at its Executive Board meeting in January 2025 [14]. Two months later, WHO’s Director-General announced a cut of over $1 billion from its 2026–27 budget, amounting to a 21% reduction of its original $5.3 billion core budget to only $4.2 billion [15]. That level of funding is wholly incommensurate with WHO’s global health mandate.

WHO’s budget cuts are leading to a dramatic organizational restructuring, including headquarters downsizing. The agency’s 10 major program divisions will be cut to only three overarching program areas: health promotion, disease prevention and control; health systems; and health emergency preparedness and response. The number of departments will be nearly halved, with health and migration among those eliminated. Health and human rights will be downgraded. Large-scale job losses are looming, expected to exceed 40% of staff, particularly at WHO’s Geneva headquarters [16].

### Shunning WHO treaties

Even after the U.S. withdrawal from WHO takes effect on January 22, 2026, the United States will technically remain a state party to the International Health Regulations (IHR), which is a distinct legally binding international agreement, also open to non-WHO member states. However, President Trump’s WHO withdrawal executive order also stated the US would not adopt or enforce the regulations’ amendments adopted in June 2024 in the wake of the pandemic and meant to facilitate equitable access to health products in a health emergency [1]. On July 18^th^, the U.S. officially withdrew from the IHR amendments in a joint statement by Health and Human Services Secretary Robert F. Kennedy, Jr. and Secretary Rubio [17]. This marks a sharp departure from the Biden administration, which was a key driver of the amendments. The president also withdrew from negotiating the WHO Pandemic Agreement, adopted by the 2025 World Health Assembly, discussed below.

### Dismantling USAID

In only a matter of weeks, and in defiance of the law, the Trump administration took USAID, the world’s largest aid agency [18], and turned it into an organization that had all but ceased to function. Secretary Rubio’s January 24 stop-work order began the rapid unravelling of USAID, with thousands of contractors laid off within a week [19]. By that point, the USAID website had been taken offline, and USAID employees were barred from entering its headquarters. In the coming weeks, the administration fired or placed on administrative leave most USAID employees [20,21].

On March 28, the Trump administration put the final nail in the coffin of USAID, ending the agency in all but name. Before President Trump entered office, USAID employed about 10,000 people. Now, the administration said, the agency would be reduced to 15 legally required positions [22].

In little over two months, America’s storied foreign aid agency was purged and effectively eliminated. On July 1, the administration officially closed the agency, folding remaining employees and programs into the State Department.

### Judicial pushback and acquiescence

On February 13, a district court judge issued a temporary restraining order requiring the government to resume foreign assistance funding. Yet, the Trump administration repeatedly refused to comply, and later that month terminated most USAID grants and contracts, asserting falsely that the terminations were the result of an individualized review of all USAID awards. The administration also asked the Supreme Court to block the judge’s order to pay grantees and contractors for work already performed. The Court, in a 5–4 ruling on March 5, directed the lower court to clarify the government’s obligations [23].

Complying with the Supreme Court’s ruling, the district court ordered the administration to pay for work already performed. It refused to block the mass termination of grants and contracts, but did require the government to obligate all appropriated foreign aid funding, an order the administration appealed [24,25].

Meanwhile, the administration took another route to avoid spending appropriated funds – through Congress. Under the Impoundment Control Act of 1974, the administration may rescind funds – refuse to spend money Congress already appropriated – if the president makes a formal request and both the House and the Senate approve within 45 days. And this is just what happened. In a special message to Congress in June, Trump proposed rescinding $8.3 billion in foreign assistance funds [26]. The following month, Congress largely acquiesced. The Rescissions Act of 2025 rescinded approximately $7.9 billion in funding for foreign aid (along with $1.1 billion for public media). A significant portion of the rescinded funds were directly related to health, including funding for global health programs, humanitarian assistance, disaster response, climate change, poverty alleviation, WHO, and UN agencies including UNICEF and the UN Development Programme [26].

Meanwhile, as litigation continued in both the district and appellate courts, the administration informed Congress at the end of August of its intent to rescind another $4 billion in foreign assistance. With fewer than 45 days before the September 30 deadline for obligating the funds, the administration would in effect run out the clock. Even if Congress did not approve, the funds would expire and no longer be available for spending [27]. On September 26, a bitterly divided Supreme Court allowed the Trump administration to rescind the $4 billion in foreign aid funding [28].

As complex litigation proceeds in the U.S., a pattern is emerging. Lower court judges are pushing back but a Republican-controlled Congress is either remaining silent or affirmatively ratifying Trump’s actions, while a super-conservative Supreme Court majority is acquiescing to the administration’s end-around the Constitution. Shattered lives will be the result.

### A new global health strategy

The *America First Global Health Strategy* discussed above will pivot away from supporting multilateral organizations and NGOs. Instead, the United States will sign bilateral agreements with governments, with a focus on commodities and frontline health workers. While HIV programs will continue in Africa, the plan shifts U.S. focus to the Western Pacific and the Americas.

Central to the plan is transitioning responsibility for health programs to partner governments, enabling the United States to reduce funding, aiming to end bilateral assistance to a majority of U.S.-supported countries in the next several years. In the short-term, partner governments will need to meet performance benchmarks to keep U.S. foreign assistance flowing. With the aim of preventing new and emerging infectious diseases from reaching the United States, surveillance and other pandemic preparedness and response activities remain a focus [4,29].

### Endangering key U.S. global health programs

Along with the devastation of foreign assistance, two key programs raise particular concerns: PEPFAR, a keystone of the global AIDS response, and sexual and reproductive health. A third area of particular concern, like sexual and reproductive health targeted by the administration, are programs supporting the health and rights of LGBTQ+ and other marginalized populations.

#### PEPFAR.

In 2023, for the first time in PEPFAR’s 20-year history, Congress failed to pass a five-year reauthorization, as some Republican lawmakers charged the program with supporting abortion [30]. Although abortions are not permitted using PEPFAR funds, a PEPFAR investigation later found that four nurses in Mozambique had done so; U.S. officials immediately took remedial measures and informed Congress [31]. Ultimately, last year Congress did pass a clean reauthorization bill, but it lasted only until March 25, 2025. Yet, March 25^th^ came and went, but Congress did not act.

In one hopeful note, however, signalling continued bipartisan support for PEPFAR, the Senate denied Trump’s request to include $400 million in PEPFAR funding cuts in the Rescissions Act of 2025, meaning that the administration is required to spend those funds as appropriated. Yet by September, it had become clear that the Office of Management and Budget was withholding several billion dollars in PEPFAR funding. Even if the funds are ultimately released, the damage has been done, with some programs forced to cut their services by 30-50% [32,33].

Furthermore, through a separate executive order, based on false claims of racial discrimination against Afrikaners, President Trump ordered a freeze on U.S. assistance to South Africa, including PEPFAR funding [34]. By one calculation, unless PEPFAR funding is replaced, more than 600,000 additional South Africans will die from AIDS by 2034 [35].

#### Sexual and reproductive rights.

In his first week, President Trump reimposed the global gag rule (Mexico City Policy), as he did in 2021, and as all Republican presidents have done since Ronald Reagan [36]. This policy precludes U.S. funding going to any foreign organization that provides abortion-related services, even with non-U.S. funds. During his first term, Trump vastly expanded the global gag rule to cover all U.S. global health funding – encompassing 20 times the funding as family planning alone [37] – including PEPFAR. His January executive order adopted this same approach.

During his first term, President Trump completely defunded the UN Population Fund (UNFPA), the UN agency tasked with improving sexual and reproductive health of women, girls, and young people. Now, it has happened again as part of the mass termination of USAID awards, with all 48 grants to UNPFA, worth $377 million, canceled [38]. Among the consequences: 9 million Afghans cut off from reproductive and maternal health services, 1,700 Afghan female health workers losing their jobs, and 600,000 Bangladeshis losing domestic violence prevention services [39]. Following a review Trump ordered through a presidential memorandum to end all funding for programs supporting coercive abortions or involuntary sterilization, long a Republican accusation of the UNFPA, the administration announced that it would not provide any further funding for the agency [40].

As is so often the case, marginalized populations will suffer most [41,42].

#### LGBTQ± and other marginalized populations.

The foreign aid freeze, review, and subsequent cuts intersect with several other executive orders that cut programs to support marginalized populations. Trump’s first-day executive orders included one targeting “diversity, equity, and inclusion” (or “diversity, equity, inclusion, and accessibility”) programs, directing all such programs to be terminated based on a baseless assertion that these are discriminatory [43]. Another executive order directed an end to all federal funding of “gender ideology” – essentially a policy of denying the existence of transgender people [44]. As a result of these orders, HIV-related and other foreign assistance programs supporting the health and rights of members of the LGBTQ+ community were prime targets for termination [45], and nearly all would be cancelled [46]. Such programs had even been expressly excluded from the foreign aid freeze humanitarian waiver [47].

### Irreparable harms

It would be comforting to think all this damage can be undone under a new U.S. president in 2029. Sadly, the damage will be enduring and often irreversible. Programs discontinued will struggle to restart, career scientists and humanitarians laid off cannot easily be replaced, and organizations that lose funding may be forced to close forever. Trust earned over decades, both with patients and with partner organizations and governments, has been shattered, and cannot easily be repaired. Ultimately, the harm will be calculated in a fatality count that will very likely rise into hundreds of thousands, at least, and the millions if the programs are not restored. It is important to reflect on the sheer scale of this American carnage.

Among the most consequential cuts will be the end of U.S. funding to Gavi, the Vaccine Alliance. This alone stands to cost 1.2 million children their lives [23]. The cuts could lead to 18 million new malaria infections a year [20]. One funding cut will leave over 100,000 people in South Sudan without quality health and nutrition, while another cut means that more than 400,000 people in Burkina Faso will lose access to clean water and child protection [48]. USAID’s storied Famine Early Warning Systems Network has ceased operating [49]. And this is all alongside children who will not be educated, people with disabilities who no longer receive services, democracy and human rights advocates who lose vital support, and more.

How deep these harms will be, how many deaths will result, depends on what happens next. In a worst-case scenario, if the deeply diminished U.S. foreign assistance programs following the mass termination of grants and contracts defines U.S. foreign assistance for the rest of this administration, 14 million people whose lives would have been saved without these cuts will instead die through 2030, including 4.5 million children under five, accordingly to one analysis [50]. Trump’s FY2026 budget request proposed cutting foreign assistance in half – a $30 billion reduction – including a 61% decrease in humanitarian assistance and 62% cut in global health funding – as well as an additional $20 billion in rescissions [51]. Congress often goes its own way with the budget, effectively ignoring the major cuts to foreign assistance Trump proposed in his first term. Will the traditional bipartisan support for these programs re-emerge, or does Congress’s approval of almost all of Trump’s proposed rescissions indicate a new reality?

Looking longer term, a new president in 2029 could reverse some of Trump’s actions, but others will take far longer to rectify. A new president could return to the United States to WHO. Since Trump unilaterally withdrew from the organization, without Congress’s approval and without paying current dues to the WHO, a condition Congress had set for WHO withdrawal [52], a new president should be able to simply declare Trump’s withdrawal null and void. A new president could also affirm U.S. acceptance of the IHR amendments and sign the Pandemic Agreement.

The destruction of U.S. foreign aid infrastructure will be much more difficult, and take longer, to undo. Health and other specialized staff will be hard to replace. Relationships built over decades – with governments, NGOs, and especially communities – once broken, are difficult to repair. A decision would be required whether to reconstitute USAID or accept its illegal closing as a fait accompli.

All this will come in a changed foreign assistance and development landscape. A new, lower norm may have been established for U.S. foreign assistance funding. A new administration and Congress would need to decide whether there are elements of the *America First Global Health Strategy* to retain, what former elements of U.S. health assistance to restore – like partnerships with NGOs and an increased focus on Africa – and what new approaches to establish. Reduced foreign assistance will have further, still unknown implications linked to the success of efforts to increase domestic health funding and capacities, and bolster regional organizations like the Africa CDC.

Of course, the most significant impacts of the administration’s actions on international health assistance and humanitarian assistance are beyond repair: the millions of people who will lose their loved ones will never get them back.

## Undermining U.S. national interests

President Trump was elected on a platform of “America First,” so a key question is whether all the evisceration of funding and staff, while disavowing international organizations and norms, will advance U.S. national interests. The answer is no. To the contrary, his actions will severely weaken U.S. national security and diplomatic influence.

U.S. health security is advanced when outbreaks are detected and contained at their source, before they reach America’s shores. This requires international partnerships. The U.S. was the world’s largest funder of WHO’s health emergencies program. WHO’s GOARN is an international response network to address health crises wherever they arise. Both will be significantly weakened. Domestic public health capabilities themselves are severely weakened. Secretary Kennedy announced on March 26 that HHS was cancelling grants to state public health agencies worth more than $11 billion [53]. And on May 2, Trump’s 2026 budget proposed a 50% cut to CDC’s budget, nearly $3.6 billion. America’s flagship public health agency will now be far less able to respond to outbreaks worldwide.

WHO’s Health Emergencies Programme can often respond to outbreaks in countries where the U.S. might not be welcome—for example, polio immunization in Afghanistan, Pakistan, and Gaza. Foreign assistance cuts, meanwhile, have terminated awards for strengthening laboratory capacities and surveillance, including in Latin America, even as “the Western Hemisphere is awash with transnational threats to human and animal health [54]”.

Withdrawal from WHO will impede access to epidemiological data and scientific information vital to U.S. national interests. The U.S. is experiencing major outbreaks of measles and H5N1 influenza in dairy cattle. And yet, CDC will not participate in WHO’s Global Influenza Surveillance and Response System (GISRS) or its Measles and Rubella Laboratory Network, consisting of over 700 laboratories serving more than 160 countries [55]. America relies on GISRS for both seasonal influenza viruses and to track novel avian strains. Yet, both WHO surveillance networks are now in danger without U.S. funding—imperilling health worldwide.

U.S. global engagement on health builds alliances. Yet again the administration’s actions are weakening U.S. values and interests. Global health assistance is a tremendous bargain for what it buys in soft power. As President Biden’s USAID Administrator Samantha Power observed, “for most of the world’s population, the investments and work of USAID make up the primary (and often only) contact with the United States.” Beyond building goodwill, it is a source of political capital, “making it more likely that when the United States makes hard requests of [other countries’] leaders…they say yes [56].” This soft power has been replaced by a sense of abandonment, a vanishing trust that will become hard to restore irrespective of future policy and funding choices.

The rapid-fire reductions in global health funding and abrupt retreat from international organizations and norms, certainly represent a grievous wound to world health. But they may inflict a still more grievous wound to the U.S. population over the long term.

## Creating opportunities out of peril

It took only weeks for President Trump to turn the U.S. from a biomedical and global health leader to a pariah, shattering trust while shattering lives, all the while posing fundamental threats to a law-based international order grounded in human rights and democratic governance. The country that had been at the core of that international order for eight decades, however imperfectly, now steps away, and even positions itself nearer to autocracies than democracies. So, how can governments and other actors respond with a force of at least equal magnitude pulling in the opposite direction? Put another way, how might the international community create unexpected opportunities out of the peril of U.S. disengagement?

There is no sugar coating the impacts of President Trump’s destructive rhetoric and policies. But the administration’s actions do create opportunities to end considerable reliance on a single country. Instead of a major retrenchment in global health precipitated by the Trump administration, the international community should instead create a more resilient, flexible system, with increased power for, capacities in, and funding from countries of the Global South. Here are several possibilities.

### Globally distributed manufacturing

COVID-19 vividly demonstrated the dangers of overly relying on high-income governments to donate and fund lifesaving vaccines and treatments. The world needs to move from a model of philanthropy to one of self-reliance. African countries, for example, are now embracing the idea of “health sovereignty,” which builds domestic and regional health system capacities. To ensure ample and affordable supplies of medical countermeasures, shared more equitably, we must diversify manufacturing capacities. Put another way, rather than waiting for charitable donations (which come too little and too late), LMICs seek to develop their own medical products. That could be a win-win: it would make historically neglected regions more self-reliant, while avoiding global supply shortages of medical products, helping everyone.

In an important step, WHO’s mRNA Technology Transfer Programme is working with at least 15 partners in Africa, Asia, and Latin America to become mRNA technology production sites, with the aim of sustainable mRNA technology production capacity across LMIC regions [57]. Much more will be needed, though, especially in Africa. There, most countries import 70% or more of their medicines [58], 90% or more of their medical devices [59], and a staggering 99% of vaccines [59]. Hardly any active pharmaceutical ingredients are produced in Africa. As of 2022, the African continent had 649 pharmaceutical manufacturing plants, whereas in 2019, China and India (broadly similar in population) had 5,000 and 10,500 such plants, respectively [59].

Last year, WHO’s regional office in Africa (WHO AFRO) adopted a framework to increase local pharmaceutical manufacturing, with targets of local production achieving 55% of market share by 2035, including at least 50% for vaccines [59,60]. The Africa CDC is also expanding its capacities and partnering with WHO AFRO. All this will require training scientists and health professionals, strengthening regulatory authorities, and technology transfer partnerships [59]. It will also require significant funding, estimated at $11 billion by 2030 for local pharmaceutical companies, along with $100 billion in infrastructure investments [61].

### Countries that can contribute must contribute

Traditional global health donors are unlikely to compensate for U.S. cuts to global health and humanitarian assistance, as they are making their own cuts. Most dramatically, the United Kingdom, which in 2020 announced it was cutting development assistance from 0.7% to 0.5% GNI [62], now plans a further reduction to 0.3% GNI to enable increased defense spending [63].

In response to the war in Ukraine, the European Union will exempt increases in defense spending from its 3.0% GNI debt ceiling [64]. The EU could do the same for development assistance, with its life and death implications. Wealthy countries that provide a small percentage of GNI to development assistance should step up—for example, in 2024, Australia and South Korea invested 0.19% and 0.21% GNI, respectively, to development assistance. The average OECD Development Assistance Committee member contribution was 0.33% [65]. The wealthy Gulf states should also increase their development assistance to meet the OECD target of 0.7% [66].

China’s development assistance amounts to less than 0.05% GNI (0.03% at the lower end) [67]. Yet China has demonstrated capacity to generate enormous resources, such as for defense and economic stimulus. Even reaching 0.20% GNI would inject about an additional $25 billion in development assistance. New funding alone, however, is not enough to ensure impact. China and other donors without established, effective bilateral health agencies with a proven track record should direct much of the global health portion of their foreign assistance to global health institutions that do have a proven track record, including WHO, the Global Fund, and Gavi.

While increased contributions would create more resilient health financing, it is just as important for LMICs to increase domestic health investments. In the Abuja Declaration in 2001, African governments pledged to spend at least 15% of their national budgets on the health sector; yet only two countries are doing so [68].

### High-income countries must offer debt relief

Governments that have saddled lower-income countries with crippling debt should embark on an ambitious program of debt relief. In 2023, the United Nations reported that countries whose populations today comprise 3.3 billion people spent more on servicing their debts than either health or education [69]. LMICs collectively spend 3.7% of their GNI servicing their debts [70]. Put another way, LMICs spend twice their official development assistance on debt payments [71,72].

In November 2020, the G20 launched its Common Framework for Debt Treatments, which was wisely designed to include all creditors – multilateral institutions and high-income countries, but also China and private creditors – but has been off to a languid start. Four years after its launch, only four of 73 eligible countries had begun the process; none had completed it [73]. Reducing nations’ debt, and thus debt service payments, will free up funds to invest in health, buffering against global shocks.

### Local capacities

Far too much of traditional foreign assistance is funnelled through large international NGOs, rather than going directly to the people. Local organizations need the capacities to stand on their own regardless of the global landscape. Development partners should accelerate localization – directly funding local NGOs, as well as increasing local actors’ power in funding-related decision-making. Strategies would include funders easing reporting requirements, increasing outreach to local organizations, welcoming funding applications in local languages [74], and increasing funding for local capacity development. Pooled funding could channel health and humanitarian funding to local organizations [75]. Another benefit: local salaries mean that significantly more may be possible for equivalent sums, enabling remarkable impact at low cost.

### WHO resilience and reforms

In this tumultuous era, strong WHO leadership is crucial. Yet the agency is chronically underfunded and could solidify the full trust and support of its members with targeted reforms to shore up institutional accountability and transparency. A first step would be to accelerate the multi-budget cycle increase in assessed contributions as a proportion of WHO’s budget [76]. In 2022–23, member states paid 22% of the organization’s base budget through assessed contributions, a proportion currently slated to increase to 50% of the 2022–23 base budget by the 2030–31 biennium. WHO should instead reach the 50% level with its 2028–29 budget, with further increases to follow. The current disruptions in core WHO operations cannot wait to be repaired until 2030. Furthermore, in the 2030–31 budget cycle and going forward, assessed contributions should be based on that biennium’s budget, rather than continue to harken back to a presumably smaller 2022–23 budget.

To regain public confidence, WHO should also improve transparency, financial oversight, and accountability. The recent WHO staff vote of no confidence in leadership was a call for good governance [77]. Among many areas for reform, WHO could create an independent inspector-general with authority to identify and investigate inefficiencies, ensure financial stewardship, and monitor and redress undue state influence and state noncompliance with the IHR.

### Global health diplomacy and the Pandemic Agreement

WHO made a powerful statement of resilience by ratifying the Pandemic Agreement at the May 2025 World Health Assembly. Indeed, the U.S. withdrawal from negotiations opened opportunities to achieve consensus on more equitable approaches that the U.S. had opposed, such as on technology transfer and access to pandemic products. And adopting the Pandemic Agreement has enormous symbolic value, signalling an imperative to forge normative and diplomatic consensus at a time of shattered international cooperation. Member states could make a still more powerful statement by rapidly negotiating a robust Pathogen Access and Benefits Sharing (PABS) system in an Annex to the treaty in 2026. The 2025 World Health Assembly charged an Intergovernmental Working Group with negotiating a PABS system. It is essential that the working group succeed because the full treaty cannot be opened for signature until the PABS system is adopted.

The PABS annex, moreover, should be adopted in an historic World Health Assembly Special Session hosted in the Global South. This meeting in the Global South should not be a one-off event, but the beginning of a new practice of holding World Health Assembly and Executive Board sessions outside Geneva, especially in the Global South. Besides its symbolic importance, this could enhance public understanding of and support for WHO.

## Solidarity forward

In tumultuous times when nationalism is rising, the instinct is to retreat inwards. Yet, it is the exact opposite that offers the best chance of rising above isolationism and financial austerity. The world can emerge from cascading crises to a healthier future. And one day, perhaps less than four years from now, a new US president may return to international engagement, but in a more equal landscape not dominated by hegemons. If solidarity is the order of the day – solidarity with people around the world, especially those who are marginalized and living in vulnerable situations – it may be possible to imagine, and construct, a global system that will create a safer, healthier, and more resilient world.
